# Immunohistochemical Evaluation of Phosphatase and Tensin Homolog (PTEN) Expression in Endometrial Lesions: A Cross-Sectional Study in a Tertiary Care Center in South India

**DOI:** 10.7759/cureus.94512

**Published:** 2025-10-13

**Authors:** Sanjana Pamidimukkala, Srismitha S, R. S. Gayathri Priyadarshini

**Affiliations:** 1 Pathology, Sree Balaji Medical College and Hospital, Chennai, IND

**Keywords:** endometrial carcinoma, endometrial lesions, immunohistochemistry staining, pten gene mutation, tumor suppressor gene

## Abstract

Background: Endometrial carcinoma is a common gynecological malignancy, with type I endometrioid carcinoma often arising from precursor lesions like endometrial hyperplasia. The tumor suppressor gene PTEN (phosphatase and tensin homolog) regulates cell proliferation and apoptosis via inhibition of the phosphoinositide 3-kinase/protein kinase B (PI3K/AKT) pathway. Its loss is considered an early molecular event in endometrial carcinogenesis. This study aimed to evaluate the immunohistochemical expression of PTEN across various endometrial lesions and correlate its expression with histopathological subtypes and tumor grade.

Methodology: This cross-sectional study was conducted in the Department of Pathology, Sree Balaji Medical College and Hospital, from July 2023 to June 2025. Fifty cases were included: disordered proliferative endometrium (*n* = 3), endometrial hyperplasia without atypia (*n* = 23), with atypia (*n* = 6), endometrial carcinoma (*n* = 17), and papillary serous carcinoma (*n* = 3). Tissue sections were stained with hematoxylin and eosin and subsequently subjected to immunohistochemical staining using a rabbit monoclonal PTEN antibody (clone QR042, IVD class, rabbit IgG isotype). PTEN expression was scored based on staining intensity (0-3) and percentage of positive cells (0-4). Statistical analysis was performed using SPSS version 26 (IBM Corp., Armonk, NY).

Results: PTEN expression was preserved in most benign lesions, with moderate to strong staining in disordered proliferative endometrium and hyperplasia without atypia. Reduced staining was seen in hyperplasia with atypia. Among endometrioid carcinomas, 10/15 (66.7%) showed complete loss of PTEN, and loss correlated with higher histological grade and deeper myometrial invasion. All papillary serous carcinomas showed complete loss of PTEN. The association between PTEN expression and histopathological diagnosis was statistically significant (χ² = 52.38; *P *< 0.001).

Conclusions: Progressive loss of PTEN expression from benign to malignant lesions highlights its potential as an early diagnostic and prognostic marker in endometrial carcinogenesis.

## Introduction

Among all invasive malignancies in women, 2.3 per 10,000 cases are attributed to endometrial carcinoma [1,2]. Endometrial hyperplasia is a recognized precursor lesion predominantly associated with type I endometrioid endometrial cancer (EEC). The primary factor implicated in the pathogenesis of endometrial hyperplasia and subsequent carcinoma is prolonged estrogenic stimulation that is unopposed by the differentiating effects of progestins [3]. This hormonal imbalance promotes endometrial proliferation and may set the stage for atypical changes. Several clinical and reproductive factors, such as obesity, granulosa cell tumors, early menarche, late menopause, and extended periods of anovulation, are established risk factors for endometrial cancer [4,5].

Two major histopathological subtypes of endometrial carcinoma have been recognized: Type I (endometrioid) and Type II (non-endometrioid). These two forms show distinct behavioral patterns and clinicopathological characteristics [4,6]. Type I endometrioid carcinomas are typically estrogen-related, often preceded by endometrial hyperplasia, and occur more frequently in perimenopausal or early postmenopausal women. In contrast, type II carcinomas, including serous and clear cell histologies, arise predominantly in older women, typically in their sixties and seventies, and are unrelated to estrogen stimulation. A substantial association exists between a history of endometrial hyperplasia or unopposed estrogen exposure and the development of type I endometrial carcinoma.

Endometrial carcinogenesis is driven by stepwise accumulation of genetic and epigenetic alterations affecting oncogenes and tumor suppressor genes. Among the critical molecular events implicated, inactivation or mutation of the tumor suppressor gene PTEN (phosphatase and tensin homolog deleted on chromosome 10) is one of the most frequent abnormalities observed in endometrial carcinoma [5, 7]. PTEN is located on chromosome 10q23.3 and functions as a key negative regulator of intracellular signaling pathways. It inhibits cell proliferation and promotes differentiation by counteracting signaling cascades activated by growth factors and integrins. The PTEN protein also participates in several cellular processes, including the regulation of angiogenesis, the induction of apoptosis in injured cells, and the modulation of cell adhesion and migration. 

Mutations or loss of PTEN function lead to constitutive activation of downstream proliferative pathways, thereby contributing to oncogenesis. Indeed, PTEN alterations have been documented not only in endometrial tumors but also in a variety of other human malignancies, including thyroid, brain, and prostate cancers [7]. In endometrial carcinoma, PTEN mutations are considered early events in tumorigenesis, often detected in atypical endometrial hyperplasia and even in histologically normal endometrial glands adjacent to carcinomas. This highlights their pivotal role in the initiation of neoplastic transformation of endometrial epithelium.

At the molecular level, PTEN exerts its tumor suppressive effects primarily by negatively regulating the phosphatidylinositol 3-kinase (PI3K)/AKT signaling pathway [5,8]. PTEN achieves this by dephosphorylating phosphatidylinositol (3,4,5)-triphosphate (PIP3), thereby reducing the availability of PIP3 for recruitment and activation of AKT (protein kinase B) at the cell membrane. Reduced AKT activation subsequently leads to diminished phosphorylation of downstream substrates involved in cell survival, growth, and proliferation. Because cellular homeostasis depends critically on balanced PI3K/AKT signaling, disruption of PTEN function removes an essential inhibitory checkpoint, fostering uncontrolled proliferation and resistance to apoptosis.

Initiation of PI3-K recruitment by activated cell surface receptors leads to phosphorylation of the phosphatidylinositol (4,5)-bisphosphate (PtdIns(4,5)P2) substrate, resulting in the generation of PtdIns(3,4,5)P3 [9-11]. This lipid product serves as a docking site for AKT protein kinase and its upstream regulator PDK1, facilitating their activation [6,9,10]. Loss of PTEN disrupts this tightly controlled signaling cascade and promotes oncogenic progression.

Given the central role of PTEN in endometrial carcinogenesis, assessing its protein expression in various endometrial lesions has gained interest. Immunohistochemical (IHC) evaluation of PTEN expression has emerged as a practical approach to detect lesions exhibiting PTEN loss, regardless of the underlying genetic or epigenetic mechanism causing PTEN dysfunction [12]. Such evaluation may help identify precursor lesions and early-stage carcinomas that are likely to have undergone PTEN inactivation. Understanding the status of PTEN expression across the spectrum of endometrial lesions can provide valuable insights into the molecular pathogenesis of endometrial carcinoma and may offer prognostic and potentially therapeutic implications.

The purpose of the present study is to evaluate the staining intensity and extent of PTEN expression in various endometrial lesions. This investigation seeks to elucidate the pattern of PTEN alterations across different stages of endometrial pathology, thereby clarifying its role in the early events of endometrial carcinogenesis.

## Materials and methods

Study design

This was a cross-sectional study undertaken to evaluate the IHC expression of the tumor suppressor protein PTEN in various endometrial lesions. The study was conducted in the Department of Pathology, Sree Balaji Medical College and Hospital, over a two-year period from July 2023 to June 2025.

Study population

The study included endometrial tissue samples obtained as either endometrial biopsies or hysterectomy specimens. These cases were selected based on histopathological confirmation. The included diagnostic categories were disordered proliferative endometrium, endometrial hyperplasia with or without atypia, endometrioid endometrial carcinoma, and papillary serous carcinoma.

Inclusion and exclusion criteria

All cases fulfilling the above diagnostic criteria and confirmed on routine histopathological examination were included. Patients with a known history of endometrial carcinoma who had previously received radiotherapy or chemotherapy were excluded to eliminate therapy-related alterations in PTEN expression. Tissues that were inadequately fixed, poorly processed, or had insufficient endometrial tissue were also excluded. Any other endometrial pathology not fitting the inclusion criteria was excluded to maintain uniformity in study groups.

Sample size

The sample size for this study was determined using standard statistical methods, incorporating assumptions to ensure adequate precision and power. A confidence level of 95% and a statistical power of 80% were considered appropriate to detect meaningful differences. The calculation accounted for an expected margin of error of 5%, a pooled standard deviation of 10.5, and an assumed intra-class correlation of 0.3 between outcome measures. Based on these parameters, the estimated minimum required sample size was 50 participants.

Data collection and clinical details

For each case, demographic and clinical data such as age, menstrual status, and presenting complaints were obtained from hospital records. Menstrual cycle phase at the time of tissue sampling was noted wherever applicable. All data were recorded in a structured proforma for subsequent correlation with the histopathological and IHC findings.

Specimen processing

All included specimens were grossed according to standard protocols, ensuring representative sections were taken from pathological areas. Tissues were fixed in 10% neutral buffered formalin for 16-18 hours to ensure adequate preservation. After fixation, tissues were processed in an automatic tissue processor (Leica Microsystems GmbH, Wetzlar, Germany), undergoing sequential dehydration through graded alcohols, clearing in xylene, and embedding in paraffin wax.

Section cutting and routine histopathology

Paraffin blocks were trimmed, and 4-µm-thick sections were cut using a Leica manual microtome for routine histopathological evaluation. These sections were mounted on standard glass slides (75 × 25 × 1.35 mm) and stained with hematoxylin and eosin (H&E). Routine H&E staining was done using standard protocols: deparaffinization in xylene, rehydration in descending alcohol grades, staining in Harris hematoxylin, differentiation in 1% acid alcohol, bluing in lithium carbonate, counterstaining with eosin, dehydration, clearing, and mounting with DPX. These slides were reviewed to confirm the histopathological diagnosis before proceeding to immunohistochemistry.

Preparation of slides for immunohistochemistry

For immunohistochemistry, 3 µm sections were cut and mounted on positively charged slides prepared using poly-L-lysine to enhance tissue adhesion. These sections were incubated at 60-70°C for 30 minutes to ensure proper fixation on the slides.

Reagents and materials for immunohistochemistry

The materials used for immunohistochemistry included an incubator, a pressure cooker with an induction stove, distilled water, Tris-EDTA (ethylenediaminetetraacetic acid) buffer (50x, pH 9) for antigen retrieval, immunowash buffer (25x) as the washing solution, and Harris hematoxylin for counterstaining. Tris-EDTA buffer was prepared by diluting one part of the stock buffer in 49 parts of distilled water. The immunowash buffer was prepared by diluting one part of the stock buffer in 24 parts of distilled water.

The primary antibody used was Rabbit Monoclonal anti-PTEN (Clone QR042, IVD class, Rabbit IgG isotype), which shows both cytoplasmic and nuclear localization. Breast, renal, and prostate carcinoma tissues were used as positive controls for PTEN. The detection system employed was the Poly Q Stain kit (PolyExcel) specific for rabbit monoclonal antibodies, which included PolyExcel H2O2, PolyExcel Target Binder, PolyExcel PolyHRP, PolyExcel DAB substrate buffer, and DAB chromogen.

Immunohistochemistry procedure

Deparaffinization was carried out using two changes of xylene for 10 minutes each, followed by hydration through descending grades of alcohol and rinsing in distilled water. Antigen retrieval was performed in a pressure cooker with Tris-EDTA buffer (pH 9) for 15 minutes (three whistles). After cooling, the slides were washed twice in distilled water and once in Tris-buffered saline (TBS).

Endogenous peroxidase activity was blocked using hydrogen peroxide (H₂O₂) for 10 minutes in a moist chamber, followed by two washes in immunowash buffer. The slides were then incubated with PolyExcel Target Binder for 12 minutes, washed twice in buffer, and incubated with PolyExcel PolyHRP for 12 minutes. After washing, the primary antibody (anti-PTEN) was applied to the tissue sections and incubated for 60 minutes at room temperature (approximately 20-25 °C) in a humidified chamber to prevent drying. Following incubation, the slides were rinsed in phosphate-buffered saline (PBS) to remove unbound antibody. Visualization was achieved using 3,3′-diaminobenzidine (DAB) chromogen for 5 minutes, resulting in a brown reaction product at sites of PTEN expression. The slides were then washed in distilled water, counterstained with hematoxylin for 15 seconds, and rinsed under running tap water for 5 minutes. The sections were air-dried, dehydrated through ascending grades of alcohol, cleared in xylene, and mounted using DPX.

Evaluation and scoring of PTEN expression

PTEN immunostaining was assessed by examining both the intensity and the proportion of positive cells. PTEN showed cytoplasmic and nuclear staining patterns. The staining intensity was graded on a 0 to 3 scale as follows: 0 = no staining, 1 = weak, 2 = moderate, and 3 = strong staining. The percentage of positive cells was categorized as 0% (negative), <10%, 10%-50%, 51%-80%, and >80% positive cells. Cases showing loss of PTEN expression were defined as those with complete absence or markedly reduced staining in the lesional cells compared to the internal control stromal cells. The scoring was performed independently by two pathologists who were blinded to the histopathological diagnosis. Discrepancies were resolved by consensus review.

Ethical considerations

The study was conducted after obtaining approval from the Institutional Ethics Committee of Sree Balaji Medical College and Hospital (002/SBMCH/IHEC/2023/2003). For archival anonymized samples, a waiver of consent was obtained from the ethics committee.

Statistical analysis

The data collected were entered into Microsoft Excel and analyzed using IBM SPSS Statistics for Windows, Version 26.0 (IBM Corp., Armonk, NY). Descriptive statistics such as mean, standard deviation, and proportions were used to summarize demographic and clinicopathological characteristics. The association between PTEN expression (intensity and percentage scores) and different histopathological categories of endometrial lesions was analyzed using the chi-square test or Fisher’s exact test, as appropriate. A *P*-value less than 0.05 was considered statistically significant.

## Results

A total of 50 cases were analyzed, with the highest proportion belonging to the 41-50 years age group (24 cases, 48.0%) and the lowest in both the 31-40 years and 71-80 years groups (3 cases each, 6.0%). Regarding specimen types, total abdominal hysterectomy (TAH) was the most common (25 cases, 50.0%), followed by endometrial sampling (22 cases, 44.0%) and vaginal hysterectomy with pelvic floor repair (3 cases, 6.0%). Histopathological diagnosis showed endometrial hyperplasia without atypia in 23 (46.0%) cases, endometrial carcinoma in 15 (30.0%) cases, endometrial hyperplasia with atypia in 6 (12.0%) cases, disordered proliferative endometrium in 3 (6.0%) cases, and papillary serous carcinoma in 3 (6.0%) cases. Among the 17 cases of endometrioid carcinoma, Grade 3 was the most frequent (11, 64.71%), followed by Grade 2 (5, 29.41%) and Grade 1 (1, 11.76%). Myometrial invasion assessment in endometrial carcinoma cases that underwent TAH revealed >50% invasion in 10 (58.8%) and <50% invasion in 7 (41.2%) cases (Table 1).

**Table 1:** Clinicopathological characteristics of the study population.

Category	Subcategory/Grade	No. of cases	Percentage
Age group (years)	31-40	3	6.0%
	41-50	24	48.0%
	51-60	11	22.0%
	61-70	9	18.0%
	71-80	3	6.0%
Types of specimens	Endometrial sampling	22	44.0%
	Total abdominal hysterectomy (TAH)	25	50.0%
	Vaginal hysterectomy + pelvic floor repair	3	6.0%
Histopathological diagnosis	Disordered proliferative endometrium	3	6.0%
	Endometrial hyperplasia w/o atypia	23	46.0%
	Endometrial hyperplasia with atypia	6	12.0%
	Endometrial carcinoma	15	30.0%
	Papillary serous carcinoma	3	6.0%
Histological grade (endometrioid Ca only)	Grade 1	1	11.76%
	Grade 2	5	29.41%
	Grade 3	11	64.71%
	Total	17	100.0%
Myometrial invasion (endometrial Ca, TAH only)	<50%	7	41.2%
	>50%	10	58.8%
	Total	17	100.0%

PTEN immunostaining assessment revealed that out of 50 cases, 13 (26.0%) showed no staining (0), 16 (32.0%) showed weak staining (1+), 17 (34.0%) exhibited moderate staining (2+), and only 4 (8.0%) demonstrated strong staining (3+) intensity on a 0-3 scale. When evaluated for the percentage of positively stained cells on a 0-4 scale, 13 (26.0%) cases showed no stained cells (0), 4 (8.0%) showed <10% positivity (1+), 12 (24.0%) showed 10%-50% positivity (2+), 18 (36.0%) showed 51-80% positivity (3+), and 3 (6.0%) showed >80% positivity (4+) (Table 2).

**Table 2:** Immunohistochemical expression of PTEN in endometrial lesions. PTEN, phosphatase and tensin homolog

PTEN staining category	No. of cases	Percentage (%)
Staining Intensity (0-3 scale)		
0 (No staining)	13	26.0
1+ (Weak staining)	16	32.0
2+ (Moderate staining)	17	34.0
3+ (Strong staining)	4	8.0
Staining Percentage (0-4 scale)		
0 (<0% cells stained)	13	26.0
1+ (<10% cells stained)	4	8.0
2+ (10%-50% cells stained)	12	24.0
3+ (51%-80% cells stained)	18	36.0
4+ (>80% cells stained)	3	6.0

The relationship between histological subtypes of endometrial lesions and PTEN expression (both intensity and percentage) is shown in Table 3. PTEN intensity showed marked variation across different histological types, with complete loss of PTEN expression (intensity 0) observed predominantly in endometrial carcinoma (10/15, 66.7%) and all papillary serous carcinoma cases (3/3, 100%), and endometrial hyperplasia with atypia, most cases showed weak to moderate intensity (1+, 2/6 cases). In contrast, endometrial hyperplasia without atypia displayed higher PTEN positivity, with most cases showing moderate to strong intensity (2+ in 13/23 and 3+ in 3/23 cases and disordered proliferative endometrium displayed weak to moderate staining (1+,1/3 cases and 2+, 2/3 cases). The percentage of cells stained for PTEN also followed a similar trend, with higher expression (≥51% cells stained) seen mainly in endometrial hyperplasia without atypia, while carcinoma and papillary serous carcinoma cases mostly showed absent or very low staining. The association between histological type and PTEN expression was found to be statistically significant (ꭓ² = 52.38; *P* = 0.0005), indicating a progressive loss of PTEN expression from benign to malignant endometrial lesions.

**Table 3:** Association between histological subtypes of endometrial lesions and PTEN expression. *Chi-square/Fisher's exact test. Statistically significant at *P* < 0.05. PTEN, phosphatase and tensin homolog

Histological type		PTEN intensity				Total	PTEN %					*ꭓ*^2^-value	*P*-value
		0	1	2	3		0	1	2	3	4		
Disordered proliferative endometrium	Count	0	1	2	0	3	0	0	2	0	1	52.38	0.0005*
Endometrial hyperplasia without atypia	Count	0	7	13	3	23	0	0	8	14	1		
Endometrial hyperplasia with atypia	Count	0	4	2	0	6	0	2	1	3	0		
Endometrial carcinoma	Count	10	4	0	1	15	10	2	1	1	1		
Papillary serous	Count	3	0	0	0	3	3	0	0	0	0		

**Figure 1:**
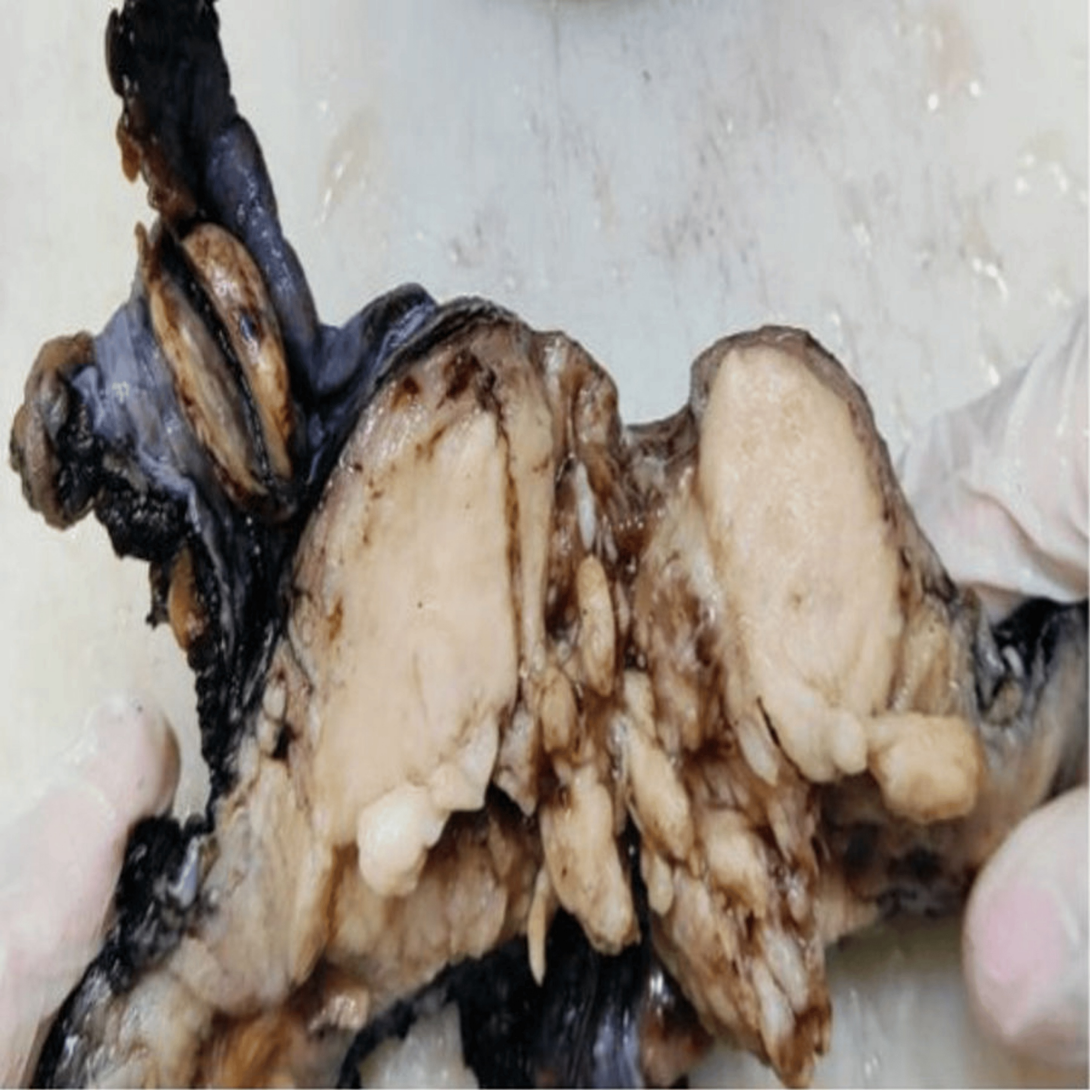
Gross appearance of endometrial cancer, with the endometrial cavity completely obscured by the tumor.

**Figure 2:**
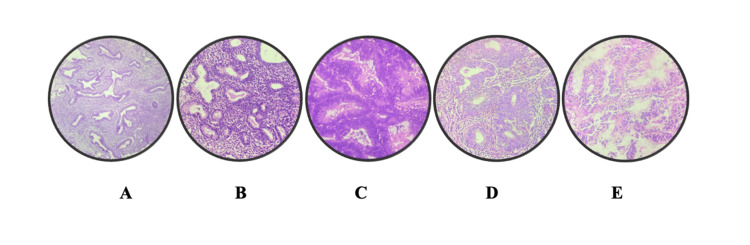
H&E images of endometrial lesions in our study: (A) disordered proliferative endometrium; (B) endometrial hyperplasia without atypia; (C) endometrial hyperplasia with atypia; (D) endometrioid endometrial carcinoma; (E) papillary serous carcinoma.

Figure 1 depicts the gross appearance of endometrial cancer, where the endometrial cavity is completely obscured by the tumor mass. Figure 2 depicts representative H&E images of endometrial lesions observed in our study: (A) disordered proliferative endometrium showing irregular glandular architecture, (B) endometrial hyperplasia without atypia characterized by glandular crowding without cytological atypia, (C) endometrial hyperplasia with atypia demonstrating nuclear atypia and architectural complexity, (D) endometrioid endometrial carcinoma with back-to-back glandular proliferation and loss of normal architecture, and (E) papillary serous carcinoma exhibiting complex papillary structures with marked nuclear pleomorphism.

**Figure 3:**
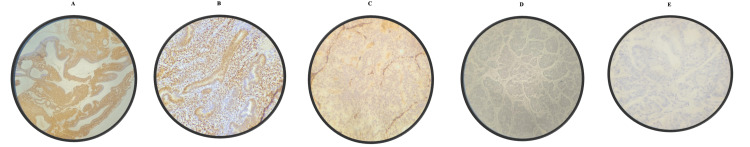
PTEN immunohistochemical (IHC) expression in disordered proliferative endometrium, endometrial hyperplasia without atypia, endometrial hyperplasia with atypia, endometrial carcinoma, and papillary serous carcinoma: (A) strong, (B) moderate, (C) weak, (D) loss, and (E) complete loss of expression. PTEN, phosphatase and tensin homolog

Figure 3 shows PTEN IHC staining in papillary serous carcinoma at 40× magnification, illustrating the spectrum of PTEN expression patterns: (A) strong, (B) moderate, (C) weak, (D) loss, and (E) complete loss of PTEN expression.

## Discussion

Endometrial cancer is the most common malignancy of the female reproductive tract, and its incidence has been steadily rising over the past two decades. This increase is largely attributed to population aging, higher obesity rates, and declining use of combined menopausal hormone therapy [13]. Unopposed estrogen exposure is a key factor driving endometrial proliferation, hyperplasia, and eventual malignant transformation. The progression from hyperplasia to carcinoma involves sequential histopathological and molecular events, among which PTEN loss is considered an early and pivotal step [14].

In our study, which included 50 cases across a spectrum of endometrial lesions, we observed a distinct age distribution. The women ranged from 31 to 80 years, with a mean age of 51 years. Nearly half of the cases were between 41 and 50 years of age, while 44% were postmenopausal. This aligns with the American Cancer Society, which notes that endometrial cancer mainly affects women over 60 years [15]. However, there is a growing trend of early-onset endometrial carcinoma (EOEC). Erkanli et al. have reported increasing prevalence of EOEC in women under 50 [16], while Setiawan et al. observed that 10%-20% of cases occur below 50 years [17]. This shift likely reflects rising metabolic risk factors, including obesity and polycystic ovary syndrome. Our finding that 42% of cases occurred in reproductive-age women supports this changing epidemiological profile.

Most patients presented with abnormal uterine bleeding, consistent with previous literature that identifies this as the hallmark symptom prompting early medical evaluation [18,19]. Because of this early presentation, many cases are diagnosed at an early stage, which contributes to the relatively favorable prognosis of endometrial carcinoma. Histopathologically, our study showed a predominance of endometrial hyperplasia without atypia (46%) and endometrioid endometrial carcinoma (30%), with fewer cases of disordered proliferative endometrium (6%), atypical hyperplasia (12%), and papillary serous carcinoma (6%). This pattern mirrors the findings of Amant et al., who also reported a predominance of hyperplastic lesions among women with abnormal uterine bleeding [20].

Among carcinoma cases, most were high grade, with 64.7% showing FIGO (International Federation of Gynecology and Obstetrics) grade 3 morphology. Although this proportion is higher than reported in some prior studies, it underscores the heterogeneity of tumor grade in different cohorts. Importantly, 58.8% of carcinoma cases in our series showed deep myometrial invasion (>50%). Myometrial invasion depth is a well-established prognostic factor, as deep invasion is strongly associated with lymphovascular space invasion, nodal metastasis, and poorer outcomes [21]. This highlights the need for careful pathological assessment to guide staging and management.

The central focus of our study was the immunohistochemical expression of PTEN, a tumor suppressor gene located on chromosome 10q23.3. PTEN antagonizes the PI3K/AKT signaling pathway, thereby regulating cell proliferation, promoting apoptosis, and maintaining genomic stability. Loss of PTEN function promotes unchecked cellular proliferation and is considered one of the earliest molecular abnormalities in endometrial tumorigenesis [11].

Our results demonstrated a progressive reduction in PTEN expression from benign to premalignant to malignant lesions. In disordered proliferative endometrium, two of three cases (66.7%) contained PTEN-null glands. This is consistent with Mutter et al., who reported PTEN-null glands in 56% of anovulatory endometrial samples, indicating that PTEN loss can occur before morphologic atypia appears. These PTEN-null glands often form clonal units within otherwise histologically normal tissue, suggesting they may act as latent precursors for endometrioid carcinoma [11].

In endometrial hyperplasia without atypia, 13 of 23 cases (56.5%) showed intact PTEN expression, while the rest showed weak or patchy staining. This finding aligns with Sarmadi et al., who reported PTEN positivity in 83.3% of similar cases [22]. Feng et al. observed a gradual decline in PTEN expression across the continuum from normal endometrium to hyperplasia and carcinoma, supporting the concept that PTEN loss is an early but not universal event [23]. The relatively preserved PTEN expression in most non-atypical hyperplasia cases suggests that additional molecular alterations may be required for progression.

In contrast, atypical hyperplasia showed a marked decline in PTEN expression, with most cases demonstrating weak (1+) to moderate (2+) staining and reduced distribution. This is consistent with Sarmadi et al., who found PTEN retention in 75% of atypical hyperplasia [22], and Lee et al., who reported 71% PTEN positivity in severe atypical hyperplasia [24]. Other studies have noted variable loss rates, but the overall trend indicates increasing PTEN inactivation as cytologic atypia emerges. These results support the idea that PTEN loss is closely linked to the transition from benign hyperplasia to premalignant atypical hyperplasia.

Most notably, the majority of carcinoma cases - particularly endometrioid and papillary serous subtypes - showed marked reduction or complete absence of PTEN expression. Only a few demonstrated weak staining in a minor proportion of cells. This mirrors the findings of Sarmadi et al., who observed PTEN loss in 48% of endometrioid carcinomas [22]. Orbo et al. and Erkanli et al. also documented frequent PTEN loss in endometrial carcinomas [16,25]. PTEN inactivation is known to promote genomic instability and resistance to apoptosis, conferring a growth advantage to malignant cells. The significant association between histological diagnosis and PTEN expression observed in our study (χ² = 52.38, *P* = 0.0005) further underscores its role in tumor progression.

Collectively, our results support a model in which focal PTEN loss begins in histologically normal or disordered proliferative endometrium, expands during atypical hyperplasia, and becomes widespread or complete in carcinoma. This stepwise loss of PTEN expression parallels the morphological progression from benign to malignant endometrial lesions. Because PTEN loss is an early and common event, it could potentially serve as a biomarker to identify women at increased risk of progression, especially those with atypical hyperplasia. Incorporating PTEN immunostaining into routine diagnostic evaluation may help stratify patients for closer surveillance or earlier intervention.

This study has certain limitations. The sample size was relatively small, with only 50 cases, which may limit the generalizability of findings. The study was cross-sectional and conducted in a single tertiary center, which may introduce referral bias. Molecular analyses such as PTEN gene sequencing or loss of heterozygosity testing were not performed; therefore, only protein expression by immunohistochemistry was assessed, which may not capture all genetic alterations. Additionally, clinical follow-up data were not available to correlate PTEN expression with patient outcomes such as recurrence or survival.

Despite these limitations, this study underscores the potential value of PTEN as a diagnostic and prognostic marker in endometrial lesions. The progressive loss of PTEN from benign to atypical hyperplasia and carcinoma suggests its utility in identifying lesions at higher risk of malignant transformation. Routine incorporation of PTEN immunostaining in the evaluation of endometrial biopsies could aid in early detection and risk stratification, especially in women with atypical hyperplasia or equivocal histology. Future studies with larger cohorts, molecular correlation, and longitudinal follow-up are warranted to validate the prognostic and therapeutic implications of PTEN loss in endometrial carcinogenesis.

## Conclusions

This study demonstrates a clear stepwise decline in PTEN expression from benign endometrial tissue through hyperplasia to carcinoma, highlighting its pivotal role in endometrial tumorigenesis. The progressive loss of PTEN immunoreactivity, especially its marked reduction or absence in high-grade carcinomas, underscores its potential utility as an early molecular marker for malignant transformation. Assessing PTEN status in endometrial lesions could aid in risk stratification, enabling earlier detection and more tailored management of patients at higher risk for progression to carcinoma.
